# Tuberculosis in HIV-Associated Cryptococcal Meningitis is Associated with an Increased Risk of Death

**DOI:** 10.3390/jcm9030781

**Published:** 2020-03-13

**Authors:** Morris K. Rutakingirwa, Fiona V. Cresswell, Richard Kwizera, Kenneth Ssebambulidde, Enock Kagimu, Edwin Nuwagira, Lillian Tugume, Edward Mpoza, Joanna Dobbin, Darlisha A. Williams, Conrad Muzoora, David B. Meya, David R. Boulware, Kathy H. Hullsiek, Joshua Rhein

**Affiliations:** 1 Infectious Diseases Institute, College of Health Sciences, Makerere University, P.O. Box 22418, Kampala, Uganda; fiona.cresswell@lshtm.ac.uk (F.V.C.); kwizerarichard@ymail.com (R.K.); kssebambulidde@gmail.com (K.S.); kanockenock@gmail.com (E.K.); lilliqntugume18@gmail.com (L.T.); edmpoza@yahoo.com (E.M.); jodobbin@googlemail.com (J.D.); coat.trial@gmail.com (D.A.W.); david.meya@gmail.com (D.B.M.); 2 Clinical Research Department, London School of Hygiene and Tropical Medicine, Keppel Street, London WC1E 7HT, UK; 3 Department of Medicine, Mbarara University of Science and Technology, P.O. Box 1410, Mbarara, Uganda; edwinnuwagira@gmail.com (E.N.); conradmuzoora@gmail.com (C.M.); 4 Department of Medicine, University of Minnesota, Minneapolis, MN 55455, USA; boulw001@umn.edu; 5 Division of Biostatistics, School of Public Health, University of Minnesota, Minneapolis, MN 55455, USA; hulls003@umn.edu

**Keywords:** Tuberculosis, cryptococcal meningitis, HIV, *Cryptococcus*, AIDS-related opportunistic infections, co-infection

## Abstract

Tuberculosis (TB) and cryptococcal meningitis are leading causes of morbidity and mortality in advanced HIV disease. Data are limited on TB co-infection among individuals with cryptococcal meningitis. We performed a retrospective analysis of HIV-infected participants with cryptococcal meningitis from 2010–2017. Baseline demographics were compared between three groups: ‘prevalent TB’ if TB treated >14 days prior to cryptococcal meningitis diagnosis, ‘concurrent TB’ if TB treated ± 14 days from diagnosis, or ‘No TB at baseline’. We used time-updated proportional-hazards regression models to assess TB diagnosis as a risk for death. Of 870 participants with cryptococcal meningitis, 50 (6%) had prevalent TB, 67 (8%) had concurrent TB, and 753 (86%) had no baseline TB. Among participants without baseline TB, 67 (9%) were diagnosed with incident TB (after >14 days), with a median time to TB incidence of 41 days (IQR, 22–69). The 18-week mortality was 50% (25/50) in prevalent TB, 46% (31/67) in concurrent TB, and 45% (341/753) in the no TB group (*p* = 0.81). However, TB co-infection was associated with an increased hazard of death (HR = 1.75; 95% CI, 1.33–2.32; *p* < 0.001) in a time-updated model. TB is commonly diagnosed in cryptococcal meningitis, and the increased mortality associated with co-infection is a public health concern.

## 1. Introduction

Tuberculosis (TB) and cryptococcal meningitis (CM) continue to be diseases of interest in the antiretroviral therapy (ART) era owing to their independent contribution to AIDS-related mortality, with TB alone contributing about 33% of the mortality in advanced HIV disease followed by cryptococcosis at 15% [1,2]. Similarly, autopsy studies in sub-Saharan Africa in hospitalized adults with HIV have attributed tuberculosis in 36%–47% of the patients and cryptococcosis in 12%–20% of the patients as the cause of death [3,4]. Wong et al. found 62% of HIV-infected patients with *Mycobacterium* infection to have had multiple infectious causes of death at autopsy, including cryptococcosis [5]. Most of the available literature on co-infection of TB in cryptococcal meningitis amongst HIV-infected patients, however, are restricted to case series and reports [6,7,8]. 

Although the local host immune response is increasingly well described in each individual infection, a paucity of data exists regarding the immunology of co-infection. Both *Cryptococcus* and *Mycobacterium tuberculosis* are intracellular pathogens that induce a type 1 immune response marked by an interaction between cells of the adaptive immune response innate immune cells like macrophages and dendritic cells. HIV depletes both the number and functionality of all immune cells resulting in impaired immune responses and a predisposition toward disseminated tuberculosis and cryptococcosis.

Despite the overlapping epidemiology, immune responses, and large influence both infections have on HIV-related mortality, co-infection of TB and cryptococcosis is not well described [8,9]. This could be attributed to the low sensitivity and specificity of the available TB diagnostic assays in patients with advanced HIV disease, resulting in missed cases of TB. This is not the case with cryptococcal meningitis, following the advent of highly sensitive and specific point-of-care cryptococcal antigen (CrAg) lateral flow assay (LFA) [10,11,12,13,14,15,16]. We aimed to describe baseline characteristics and clinical outcomes of patients with HIV-associated cryptococcal meningitis and concurrent treatment of tuberculosis.

## 2. Materials and Methods

### 2.1. Study Population

We performed a retrospective study of patients with HIV who participated in two multisite randomized clinical trials of cryptococcal meningitis in Uganda and South Africa. These trials were the cryptococcal optimal ART timing (COAT) and the adjunctive sertraline for the treatment of HIV-associated cryptococcal meningitis trial (ASTRO-CM) [17,18,19]. The COAT trial, which enrolled participants in Cape Town, South Africa, and Kampala and Mbarara, Uganda, from November 2010 to October 2012 investigated the optimal timing of antiretroviral therapy (ART) initiation after cryptococcal meningitis [17]. The ASTRO-CM trial, which enrolled participants in Kampala and Mbarara, Uganda, from August 2013 to May 2017, investigated adjunctive sertraline for the treatment of cryptococcal meningitis [18,20].

Participants in both cohorts were HIV seropositive, aged ≥18 years with a first episode of cryptococcal meningitis who gave written informed consent to participate in the studies. Study participants in COAT were followed up for 1 year, whereas those in ASTRO-CM were followed up for 18 weeks. All study participants received antifungal therapy per locally accepted standards of care, which at the time of both studies included induction therapy with amphotericin (0.7–1 mg/kg/day) and fluconazole 800 mg daily for 14 days followed by consolidation therapy with fluconazole 800 mg daily for ~4 weeks, then 400 mg through 10–12 weeks. Secondary prophylaxis was given with fluconazole 200 mg daily through at least 1 year. Participants in ASTRO-CM received adjunctive sertraline (200–400 mg daily) or placebo in addition to standard care [18]. ART was initiated 4–6 weeks after being diagnosed with CM in ART-naïve participants in ASTRO-CM and in the control arm of COAT. ART was initiated 1 week after meningitis diagnosis in participants randomized to the early-ART arm in COAT [17,18]. Institutional review boards at each participating site and the University of Minnesota approved each of these studies.

### 2.2. Study Procedures

Depending on when TB treatment was initiated, we classified the participants into three baseline categories: (1) ‘prevalent TB’ if TB was diagnosed and antitubercular treatment started more than 14 days prior to CM diagnosis, (2) ‘concurrent TB’ if antitubercular treatment was started ± 14 days from CM diagnosis, or (3) ‘No TB’ by study day 14 (after CM diagnosis). We summarized and compared baseline characteristics across the three groups.

**Figure 1:**
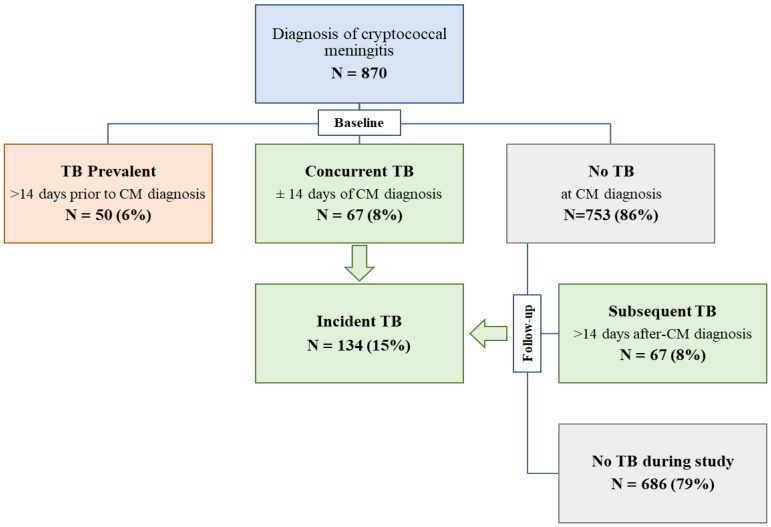
Classification of study participants. Patients with cryptococcal meningitis were classified at baseline (± 14 days of cryptococcal meningitis diagnosis) as ‘TB prevalent’, ‘concurrent TB’, or ‘no TB’. Those developing TB during study follow-up period were classified as ‘subsequent TB’. Incident TB included those with concurrent or subsequent TB. Abbreviations: CM, cryptococcal meningitis; TB, tuberculosis.

Participants who were diagnosed with TB and started on anti-TB treatment after study day 14 were classified as ‘subsequent TB’. We defined incident TB to include participants in the concurrent TB and subsequent TB groups (Figure 1). Finally, we assessed TB diagnosis as a risk factor for death.

The TB diagnosis and decision to begin TB treatment was primarily clinical, based on the presence of constitutional and clinical symptoms suggestive of TB, along with supportive findings on clinical examination, radiological imaging, microscopy or Xpert MTB/RIF when available. TB diagnostics were performed at physician discretion based on clinical suspicion and the possibility of obtaining a suitable clinical sample for testing. TB diagnoses were categorized as microbiologically confirmed, presumptive (based on radiographic findings and treatment response), or suspected diagnosis given empiric therapy. The certainty of ‘prevalent TB’ diagnoses was initially very poor in 2010, but it improved over time with increasing availability of Xpert MTB/RIF testing. 

### 2.3. Statistical Analysis

We expressed continuous variables as medians with interquartile ranges (IQRs) and categorical variables as proportions. We compared continuous variables with Kruskall–Wallis and categorical variables with chi-squared tests. As a post hoc analysis with three groups, we considered a *p* value of <0.01 as statistically significant. We used time-updated proportional hazards regression models to assess TB co-infection as a risk factor for death. For sensitivity analyses, we both included and excluded patients who were prevalent for TB at cryptococcal meningitis diagnosis. All models that included those with prevalent TB were adjusted for time on TB medications at cryptococcal meningitis diagnosis. With the time-updated models those with prevalent TB were considered to have TB co-infection on day 1, while those with concurrent or subsequent TB were considered to have TB co-infection on the day that TB medications were initiated. The fully adjusted models were adjusted for age, antiretroviral therapy (ART) status, Glasgow coma scale (GCS) <15, and cerebrospinal fluid (CSF) quantitative cryptococcal culture. 

## 3. Results

Over the study period, 870 patients with HIV and a first episode of cryptococcal meningitis were enrolled into COAT and ASTRO-CM clinical trials. At baseline, 6% (50/870) were TB prevalent, 8% (67/870) had concurrent TB, and the remaining 86% (753/870) had no TB by day 14 after meningitis diagnosis (Table 1). At baseline, 46% (n = 23) of the TB prevalent and 18% (n = 12) of the concurrent TB had records of microbiological confirmation of TB.

The median age for all participants was 35 years (IQR 29 to 40) with no differences in gender distribution, baseline Glasgow coma scale, or initial quantitative cryptococcal cultures. Patients in all three groups were severely immunosuppressed, with CD4^+^ T-cell counts <50 cells/mm^3^. The median weight in the concurrent TB group was 58 kg (IQR 50 to 60) compared to 50 kg (IQR 45 to 55) in TB prevalent and 52 kg (IQR = 48, 60) in the no TB groups (*p* < 0.001). More individuals (60%) in TB prevalent group were receiving ART at enrollment, compared with 27% in the concurrent TB group and 40% in the no TB group (*p* < 0.01). Patients with prevalent TB had lower median CSF opening pressures of 210 mmH_2_O (IQR 155 to 305) compared with patients who had concurrent TB of 260 mmH_2_O (IQR 180 to 360) and no TB by study day 14 of 280 mmH_2_O (IQR 180 to 420) (*p* < 0.01).

The median (IQR) duration on TB treatment for individuals in the prevalent TB group was 41 (29 to 72) days. Of the concurrent TB group, 15 participants had been on TB treatment for a median (IQR) of 4 (0 to 11) days, and 52 participants started TB treatment a median of 7 (2 to 11) days after cryptococcal meningitis diagnosis (Table 2). Of the 753 participants with no TB diagnosis at baseline, n = 67 (8.9%) started TB treatment a median of 41 (22 to 69) days after cryptococcal meningitis diagnosis.

Simple mortality percentages at 18 weeks did not differ among the baseline TB groups: 50% (25/50) in the TB prevalent group, 46% (31/67) in the concurrent TB group, and 45% (341/753) in the group with no TB by study day 14 (*p* = 0.81). When considering TB co-infection in a time-updated model, TB co-infection had an increased risk of mortality by 18 weeks with hazard ratio (HR) 1.62 (95% CI 1.23, 2.14; *p* < 0.001) when the TB prevalent participants were included in the model, and HR 1.72 (95% CI, 1.25, 2.36; *p* < 0.001) when the TB prevalent participants were excluded from the model, see Table 3. The risk was similar after adjusting for age, ART use, baseline Glasgow coma scale score, and baseline fungal burden, which are known mortality risk factors in cryptococcal meningitis [20,21].

## 4. Discussion

This study has demonstrated that TB is commonly diagnosed among patients with HIV-associated cryptococcal meningitis and that co-infected patients are at a higher risk of death compared to patients with cryptococcal meningitis and no TB. We found that one in five (184/870, 21%) of study participants with cryptococcal meningitis received contemporaneous treatment for TB, including 15% (134/870) who were started on antituberculosis therapy during or after cryptococcal meningitis diagnosis. This high proportion of cryptococcal meningitis patients clinically diagnosed with TB co-infection occurred during an era when there was no systematic or routine screening for TB in this severely immunocompromised population in this setting. Xpert MTB/RIF roll out began in 2011, and the perpetual challenge of obtaining sputum samples in critically ill patients with wasting in the absence of sputum induction facilities or accessing extrapulmonary samples has limited its impact in HIV-associated TB. Smear microscopy has limitations in terms of sensitivity, and culture is not routinely available, so the majority of TB diagnoses were made on clinical suspicion during this study era. Furthermore, this study completed prior to the availability of urine TB lipoarabinomannan (TB-LAM; Alere) in 2018; thus, systematic screening for TB with urine TB-LAM was not possible. One fundamental question is whether confirmed TB is under-diagnosed or empiric diagnosis of TB is over-diagnosed. 

We postulate that the introduction of systematic screening of TB using sensitive and specific TB diagnostics in severely immunosuppressed patients with AIDS will increase the microbiological confirmation of TB co-infection and could increase the overall proportion of hospitalized patients with AIDS who have confirmation of a TB diagnosis. Urine TB-LAM is an affordable and rapid point-of-care test that has a sensitivity of 45% and specificity of 92% in patients with severe HIV-related immunosuppression (CD4 < 200 cells/mm^3^) in South Africa [22,23]. With these performance characteristics in a population with 21% disease prevalence, the positive predictive value of a positive urine TB-LAM would be 59% and negative predictive value of 86%. Additional TB diagnostics have emerged since 2017 with Xpert Ultra (Cepheid, Sunnyvale, CA) and SILVAMP TB-LAM (Fujifilm, Tokyo, Japan), which should improve TB detection. Early identification and treatment of TB in this population of patients co-infected with cryptococcal meningitis would likely have a survival benefit. 

Conversely, some patients may have empiric TB misdiagnoses with missed opportunities for earlier detection of pulmonary cryptococcosis or early disseminated infection. We found among the TB prevalent group, only 60% had initiated ART at time of cryptococcal meningitis diagnosis, none had received cryptococcal antigen screening, and only 46% had a confirmed TB diagnosis. Individuals in this group would presumably have sought health care following respiratory symptoms, and thus in the absence of confirmed TB, patients could have received additional earlier diagnostic work up for other conditions beyond TB. Pulmonary fungal infections (e.g., cryptococcosis, histoplasmosis) can present as a similar subacute pulmonary illness with B-symptoms [24,25]. Thus, without CrAg screening at ART initiation, pulmonary cryptococcosis can be misdiagnosed as pulmonary TB with the exception of individuals with limited pulmonary cryptococcal involvement who may have a negative serum CrAg [26]. This is especially true in high TB prevalent settings where an anchoring bias will favor TB diagnosis, even when the available microbiologic tests are negative. Thus, a misdiagnosis of pulmonary TB may result in diagnostic delays, dissemination of cryptococcal infection, and late presentation with cryptococcal meningitis with 50% mortality. TB control programs do not consider fungal infections, and even though there is guidance on offering CrAg testing and urine LAM testing for those with advanced HIV disease, implementation of this guidance remains poor in low-resource settings [27,28].

While the mortality rates were similar for the baseline TB categories (prevalent, concurrent, or no TB), we did find with time-updated models that the risk of death by 18 weeks was significantly higher for those with incident TB co-infection. There are several possible alternate explanations for this finding. First, a possible explanation is that the sickest cryptococcal meningitis patients are started on anti-TB treatment in extremis if they are failing to improve or deteriorating clinically. Second, early co-administration of antitubercular medications and anti-cryptococcal antifungals could increase the risk for medication-related toxicity, although no overt hepatotoxicity was observed [29]. Third, there is drug–drug interaction between rifampicin and fluconazole, with induction of the cytochrome P450 system warranting dose adjustment [30]. Fluconazole doses were routinely increased by 50% with concomitant rifampicin. Fluconazole inhibits the CYP3A4 system; thus, further drug–drug interactions are possible. 

Our results must be interpreted with caution. We acknowledge the problematic nature of diagnosing extrapulmonary or disseminated TB; thus, lack of TB diagnostics for this likely resulted in a bias towards over-diagnosing extrapulmonary or disseminated TB. In many low-income settings, as in other parts of the world where TB is highly endemic, there is often a low threshold for starting TB medications in the context of clinical worsening. Thus, it might not be a surprise that those diagnosed with concurrent TB would have a higher mortality. Without the ability to confirm extrapulmonary TB with severe but relatively non-specific clinical presentations in this study, it is possible that the increased mortality we observed could have been explained through worsening cryptococcal disease or other concurrent unidentified opportunistic infections rather than TB. This highlights the need for sensitive and specific TB screening diagnostics to confirm the presence of TB co-infection in hospitalized persons with AIDS.

Based on the findings from this study, we recommend that all patients diagnosed with cryptococcal meningitis with or without symptoms suggestive of TB be aggressively screened for active TB co-infection with the best available TB diagnostic tools (urine TB-LAM, Xpert MTB/RIF, chest imaging), and antituberculosis treatment commenced when active TB is identified. Secondly, studies to evaluate the efficacy of isoniazid preventive therapy (IPT) in cryptococcal meningitis are required since international guidance is currently lacking in this population. IPT has been shown to reduce the incidence of TB in HIV-positive persons [31] and to prevent TB specifically in critically ill hospitalized HIV-infected patients with advanced immunosuppression (the same population as those with cryptococcal meningitis). To date, there has been a hesitancy in initiating IPT after CM given the possibility of active TB (and a corresponding risk of under treatment with IPT) being “hidden” by symptoms of cryptococcal disease and an inability to perform a reliable symptom screen. Clinical trials must be conducted to determine if these concerns are warranted.

## 5. Conclusions

In conclusion, we have noted that clinical TB diagnosis is a common co-infection among patients with HIV-associated cryptococcal meningitis. Our study suggests that co-infection with TB appears to be a risk factor for mortality in cryptococcal meningitis, though the reasons for this observation remain poorly understood. Under half of the TB was microbiologically confirmed; thus, improved TB diagnostics are still needed, particularly for extra-pulmonary disseminated TB. Additional studies are needed to validate our findings, investigate immune interactions at the cellular level, and to evaluate the role of TB preventative therapy, the optimal timing of anti-TB drugs, and the drug interactions and pharmacokinetics occurring among individuals co-infected with TB, *Cryptococcus*, and HIV.

## Figures and Tables

**Table 1:** Baseline characteristics of the study population.

Characteristic	TB Prevalent	Concurrent TB	No TB	*p* value *
	(>14 days Prior)	(Days −14 to +14)	(By day +14)	
	(n = 50)	(n = 67)	(n = 753)	
**Demographics**				
Age, years	35 (29, 40)	35 (30, 40)	35 (30, 41)	0.88
Women	22 (44%)	21 (31%)	312 (41%)	0.24
**Clinical characteristics**				
Weight, kg	50 (45, 55)	58 (50, 60)	52 (48, 60)	**<0.001**
Glasgow Coma Score < 15	14 (28%)	23 (34%)	303 (40%)	0.16
Receiving HIV therapy	30 (60%)	18 (27%)	298 (40%)	**<0.01**
Duration of HIV, months	3.0 (1.5, 22.3)	6.0 (0.4, 55.1)	4.1 (0.2, 37.0)	0.55
CD4 T cells/mm^3^	26 (11, 54)	21 (6, 65)	17 (7, 51)	0.25
Confirmed TB	23 (46%)	12 (18%)		
**Baseline CSF parameters**				
*Cryptococcus* culture, log_10_ CFU/mL	4.2 (2.4, 5.3)	4.4 (2.7, 5.5)	4.7 (3.3, 5.4)	0.17
Sterile CSF culture	3 (6%)	4 (6%)	51 (7%)	0.95
CSF opening pressure, mm H_2_O	210 (155, 305)	260 (180, 360)	280 (180, 420)	**<0.01**
CSF white cell ≥5 cells/mm^3^	19 (42%)	28 (44%)	287 (40%)	0.71
CSF protein, mg/dL	54 (30, 110)	71.5 (24, 164)	60 (25, 120)	0.61

Data are median (IQR) or n (%). Abbreviations: TB, tuberculosis; CSF, cerebrospinal fluid. * *p*-value from Kruskall–Wallis test for medians, Chi-squared test for proportions. Bold denotes *p* < 0.05.

**Table 2:** Timing on antitubercular medication versus cryptococcal meningitis diagnosis.

Days on TB Medications	TB Prevalent	Concurrent TB	No TB
	(>14 days Prior)	(Days −14 to +14)	(By day +14)
	(n = 50)	(n = 67)	(n = 753)
**Prior to Cryptococcal Diagnosis**			
Max	180	12	
Median (IQR)	41 (29, 72)	4 (0, 11)	
Min	17	0	
N	50	15	
**After Cryptococcal Diagnosis ^1^**			
Max		15	126
Median (IQR)		7 (2, 11)	41 (22, 69)
Min		1	12
N		52	67

Abbreviations: TB, tuberculosis; CM, cryptococcal meningitis; IQR, interquartile range. ^1^ For those with no TB history at screening, this is N and time to TB incident.

**Table 3:** Mortality risk of TB co-infection in cryptococcal meningitis in time-adjusted models compared to no TB diagnosis.

	Unadjusted Model		Adjusted Model *	
Event	HR (95% CI)	*p* value	HR (95% CI)	*p* value
**Including TB prevalent ^†^ (n = 870**				
Death by day 30	1.33 (0.90, 1.97)	0.15	1.47 (1.00, 2.17)	0.05
Any death	1.62 (1.23, 2.14)	<0.001	1.75 (1.33, 2.32)	<0.001
**Excluding TB prevalent (n = 805)**				
Death by day 30	1.30 (0.80, 2.11)	0.29	1.34 (0.83, 2.19)	0.23
Any death	1.72 (1.25, 2.36)	<0.001	1.77 (1.28, 2.43)	<0.001

* Adjusted for age, ART use, Glasgow coma scale score < 15, and CSF quantitative cryptococcal culture. ^†^ Those with prevalent TB have the time-updated indicator for TB active on study day 1.
